# Conformational transitions in human translin enable nucleic acid binding

**DOI:** 10.1093/nar/gkt765

**Published:** 2013-08-26

**Authors:** Laura Pérez-Cano, Elad Eliahoo, Keren Lasker, Haim J. Wolfson, Fabian Glaser, Haim Manor, Pau Bernadó, Juan Fernández-Recio

**Affiliations:** ^1^Joint BSC-IRB research programme in Computational Biology, Barcelona Supercomputing Center (BSC), Jordi Girona 29, Barcelona 08034, Spain, ^2^Department of Biology, Technion-Israel Institute of Technology, Haifa 32000, Israel, ^3^Blavatnik School of Computer Science, Raymond and Beverly Sackler Faculty of Exact Sciences, Tel Aviv University, Tel Aviv 69978, Israel, ^4^Bioinformatics Knowledge Unit, The Lorry I. Lokey Interdisciplinary Center for Life Sciences and Engineering, Technion-Israel Institute of Technology, Haifa 32000, Israel and ^5^Centre de Biochimie Structurale, INSERM U1054, CNRS UMR 5048, Université Montpellier 1 and 2, F-34090 Montpellier, France

## Abstract

Translin is a highly conserved RNA- and DNA-binding protein that plays essential roles in eukaryotic cells. Human translin functions as an octamer, but in the octameric crystallographic structure, the residues responsible for nucleic acid binding are not accessible. Moreover, electron microscopy data reveal very different octameric configurations. Consequently, the functional assembly and the mechanism of nucleic acid binding by the protein remain unclear. Here, we present an integrative study combining small-angle X-ray scattering (SAXS), site-directed mutagenesis, biochemical analysis and computational techniques to address these questions. Our data indicate a significant conformational heterogeneity for translin in solution, formed by a lesser-populated compact octameric state resembling the previously solved X-ray structure, and a highly populated open octameric state that had not been previously identified. On the other hand, our SAXS data and computational analyses of translin in complex with the RNA oligonucleotide (GU)_12_ show that the internal cavity found in the octameric assemblies can accommodate different nucleic acid conformations. According to this model, the nucleic acid binding residues become accessible for binding, which facilitates the entrance of the nucleic acids into the cavity. Our data thus provide a structural basis for the functions that translin performs in RNA metabolism and transport.

## INTRODUCTION

Translin, an RNA- and DNA-binding protein, was discovered in extracts of human and mouse cells and shown to assemble into a multimeric complex (1–3). The protein TRAX is a translin paralog found to be specifically associated with translin (4), and also binds single-stranded oligonucleotides when in complex with translin (5). Both translin and TRAX are highly conserved throughout eukaryotic evolution. Thus, homologs of both proteins are found in many eukaryotes, from *Homo sapiens* to *Drosophila* to the fission yeast *Schizosaccharomyces pombe* (6).

Translin and the related protein TRAX appear to be multifunctional. Based on its preferential affinity for specific sequences of single-stranded DNA flanking chromosome translocations as well as single-stranded microsatellite repeats and G-strand telomeric repeats, translin has been proposed to be involved in different functions of DNA metabolism (7–9). More importantly, the interaction of translin and translin–TRAX complexes with RNA might be involved in the control of mRNA translation and transport (2,10–14). More recent studies provide evidence for the involvement of translin–TRAX complexes in RNA interference (15–18) and tRNA processing (19). In summary, translin and translin–TRAX complexes play an important role in eukaryotic cell metabolism involving the processing of single-stranded nucleic acids.

Although dimers of translin were proposed to be the minimal unit that can bind single-stranded RNA and DNA *in vitro* (20), biochemical studies have shown that the major binding configuration of the protein is a homo-octamer (7). The first detailed electron microscopic (EM) analyses of recombinant translin suggested that it formed octameric ring structures with eight-fold (C8) symmetry axis and a central open channel that was suggested to accommodate single-stranded oligonucleotides (21,22). In their quaternary organization and subunit dimerization, these proposed translin rings appeared to be similar to hexameric ring helicases (23,24). However, x-ray crystallography analyses of the virtually identical mouse and human translin (99% sequence identity) revealed a different octameric assembly, consisting of a dimer of tetramers with two symmetry axes: one two-fold (C2) axis relating the two tetramers, and one four-fold (C4) axis relating the four dimers (25,26). The human translin–TRAX complex also showed a similar X-ray structure, with the same dimer of tetramers architecture in which two protomers of the octameric translin had been exchanged by TRAX (17). Interestingly, the recently published crystallographic structure of *Drosophila* translin showed the same asymmetric assembly observed in human translin–TRAX (27). However, in a different study, EM reconstructions of *Drosophila* translin–TRAX, while keeping the overall dimer of tetramers organization, revealed significant conformational rearrangement with a large expansion from the X-ray structure (18).

Our previous work identified and mapped oligonucleotide-binding interfaces on individual subunits of pure translin octamers (28). These interfaces were located within a large internal cavity in the crystallographic structure of translin, which in principle could accommodate nucleic acids. However, such cavity is not accessible for nucleic acid binding in the crystallographic structure, while it would be accessible in other octameric configurations such as the ones suggested by the translin and translin–TRAX EM data (18,22). Thus, several key questions regarding the structural basis of translin function remain open. Which of the above described translin octameric structures correspond to the stable translin configuration in solution? Are any of these structures able to bind nucleic acids? Are there other conformational rearrangements of translin which could allow the functional binding of nucleic acids?

In an attempt to furnish answers to these questions, we have performed an integrative study that provides new functional structures for translin and translin–RNA complexes in solution. In this work, we combined small-angle X-ray scattering (SAXS) measurements and site-directed mutagenesis experiments with computational approaches. Our data demonstrate that the configuration of translin and translin–RNA complexes in solution is a dimer of tetramers, which undergoes conformational transitions that had not been previously identified. This conformational heterogeneity clarifies the mechanism for nucleic acid binding by translin and provides a structural and dynamic picture that explains its biological functions.

## MATERIALS AND METHODS

### Preparation of recombinant human translin

A translin-encoding plasmid was transfected into the *Escherichia coli* XL1 bacteria. Translin was purified from the transformed bacteria as described (8) with some modifications. Briefly, a bacterial culture of 200 ml was grown to late log phase in LB medium containing 0.05 mg/ml of ampicillin. At this stage, isopropyl-β-d-thiogalactopyranoside (IPTG) was added at 2 mM and the culture was further incubated for 3 h. The bacteria were centrifuged, and the bacterial pellet was resuspended in 10 ml of phosphate buffer (3.4 mM NaH_2_PO_4_, 46.6 mM Na_2_HPO_4_, 300 mM NaCl; pH 8.0) containing 25 mM imidazole and complete EDTA-free mini protease inhibitor cocktail tablets (Roche), and the bacteria were lysed in a French pressure cell press. The lysate was centrifuged and the supernatant was purified in a Ni-agarose column, as described (8) with the following modifications: after loading of the lysate, the column was washed successively with 30 ml of phosphate buffer containing 25 mM imidazole and with 12 ml of phosphate buffer containing 45 mM imidazole. Then, translin was eluted in 5 ml of phosphate buffer containing 250 mM imidazole. The purified translin was dialyzed against 1000 ml of 100 mM NaCl, 10 mM Tris-Cl pH 8.0, 0.1 mM EDTA, 2 mM DTT and 5% glycerol. The molecular weight of the purified translin protomer is 26.8 KDa, corresponding to 240 aminoacids including 12 from the His-tag. The concentration of translin was determined by Coomassie staining using Coomassie Protein Assay Reagent (Bio-Rad) against BSA standard. Alternatively, the concentration of the protein was determined by measurements of absorption of UV light at a wavelength of 280 nm using Nanodrop. The protein concentrations were calculated from the absorption values using the corresponding extinction coefficient of 16 750 M^−^^1^cm^−^^1^ at 280 nm (8). The concentrations of translin determined by the staining procedure and by UV absorption measurements were found to be equivalent (±10%).

### Site-directed mutagenesis

Mutations in plasmids encoding translin were generated by using the QuikChange site-directed mutagenesis kit (Stratagene, La Jolla, CA). The sequence of the whole translin gene was determined in all mutants to ensure that mutations only occurred in the desired locations. Oligonucleotides were purchased from Sigma and from MWG-Biotech AG. The plasmid expressing translin was kindly sent to us by Dr M. Kasai from the Department of Immunology, National Institutes of Health, Tokyo, Japan. This construct was made by cloning the translin cDNA into the plasmid pQE-9 (Qiagen), such that the recombinant protein expressed from this plasmid in *E.coli* bacteria includes a His_6_ tag at the N terminus (8).

### Glycerol gradient centrifugation

Recombinant translin was loaded on a 10 ml linear 20–40% (v/v) glycerol gradient containing 10 mM Tris–HCl, pH 8.0, 100 mM NaCl, 1 mM EDTA and 2 mM DTT. The tubes were centrifuged for 24 h in a Beckman ultracentrifuge in the SW-41 rotor at 38 000 rpm at 8°C. Fractions of 0.22 ml were collected from the bottom of the gradient. The position attained by the protein was determined by SDS–PAGE of aliquots withdrawn from each fraction, followed by Coomassie blue staining of the gel. Estimations of the sedimentation constant and the molecular weight of translin were obtained by comparison with sedimentation constants of known protein markers, as previously described (29,30). The glycerol gradient centrifugation experiments performed on translin wild-type confirm the monodispersity of the preparation corresponding to an octameric arrangement.

### Small-angle X-ray scattering experiments

The SAXS measurements for translin and translin–RNA complex were carried out at the EMBL BioSAXS beamline X33 at the DORIS Storage Ring, DESY (Hamburg, Germany) using an X-ray wavelength of 1.54 Å and a sample-to-detector distance of 2.7 m. Translin samples were measured at 10°C at concentrations of 5.9, 4.1, 3.0, 1.5 and 0.8 mg/ml in a buffer containing 10 mM Tris–HCl, pH 8.0, 0.1 mM EDTA, 1 mM DTT, 5% glycerol and 100 mM NaCl. Translin–RNA complexes were prepared by mixing in the same buffer translin octamers with the RNA oligonucleotides at a stoichiometric ratio of 1:1.5, followed by incubation at 25°C for 20 min. SAXS measurements of these complexes at protein concentrations of 5.9, 3.0 and 1.5 mg/ml were also carried out at 10°C. We have previously found that by following the above mentioned incubation at 25°C, cooling of translin–nucleic acid complexes to 10°C did not affect the stability of the complexes (data not shown). The scattering patterns of the corresponding buffer solutions were recorded before and after the measurements of the protein sample. The scattering profiles measured covered a momentum transfer range of 0.0087 < *s* < 0.60 Å^−^^1^. Inspection of the consecutive 30-s X-ray exposures discarded the presence of radiation damage. No signature of aggregation was observed in any of the curves. In addition, no noticeable concentration effects were observed in the datasets indicating the absence of oligomeric equilibrium (Supplementary Figure S1). Inspection of the low-angle region of the curves indicates the presence of attractive interparticle interactions (Supplementary Figure S1) that were minimized when merging the curves (see below). Final curves at each concentration were derived after the averaged buffer patterns were subtracted from the protein ones using standard protocols with PRIMUS (31). Analyzed curves for translin and translin–RNA samples were obtained by merging the curves at different concentration using standard protocols to keep the maximum sensitivity while avoiding inter-particle interactions. Molecular weight estimations for translin and translin–ssRNA from SAXS data were performed using Porod’s volume approach and the program SAXS-MoW (32). From Porod’s volume, molecular weight estimates for translin and translin–ssRNA were 253 and 270 KDa, respectively, using a value of 1.6 for transformation. When using SAXS-MoW up to 0.3 Å^−^^1^ MW estimates of 261 and 273 KDa were obtained for translin and translin–ssRNA, respectively. In all these cases the MW is overestimated with respect to the theoretical one for a translin octamer (220 KDa). This disagreement can be explained by the fact that translin is not a globular protein but a hollow sphere. The forward scattering, *I(0)*, and the radius of gyration, *R_g_*, were evaluated using the Guinier's approximation (33), assuming that at very small angles (*s* < 1.3/*R_g_*), the intensity can be well represented as I(*s*)=*I(0)* exp(−(*sR_g_*)^2^/3). The maximum particle dimensions, D_max_, and the pair-wise distance distribution function, *p(r)*, were computed with the program GNOM (34) using a momentum transfer range of 0.020 < *s* < 0.30 Å^−^^1^. The agreement between the SAXS curve and the crystallographic structure was evaluated with CRYSOL (35) using a momentum transfer range of 0.020 < *s* < 0.40 Å^−^^1^.

### Modeling of full-length translin

The N-terminal histidine-tag and the C-terminal tail were absent from the human translin crystal structure; hence, they were assumed to be disordered in solution. We used the program RanCh (36) to calculate 10 random conformations for the N-terminal histidine-tag and C-terminal tail of 12 and 11 residues, respectively, per each of the eight subunits of the crystallographic structure (PDB 1J1J). We used these sets of conformations to reconstruct these terminal tails for all assemblies calculated along the present study by superimposing the Cα atoms of the individual tails onto the equivalent ones in each corresponding translin subunit.

### Translin conformational sampling by normal mode analysis

We have explored the conformational space of human translin (PDB code 1J1J) based on normal mode analysis. Normal Mode Analysis (NMA) assumes that the protein structure is a system of coupled harmonic oscillators connecting the atoms (to simplify calculations, only Cα atoms are often considered). The dynamics of such a system is constituted by the normal modes, each one involving all the particles of the system. Based on the normal modes that produce the most relevant conformational movements (usually the lowest frequency ones), taken either as single modes or in combination, one can reproduce (at least partially) the conformational heterogeneity in a protein. For this, we built translin models by combining three different NMA-based approaches, to explore more extensively the conformational space of translin. All NMA calculations started from the Cα coordinates of translin, excluding the unstructured N-terminal histidine-tag and C-terminal tail. First, we used elNémo server (http://www.igs.cnrs-mrs.fr/elnemo/) (37) to exhaustively obtain displacements using the five lowest-frequency modes, either as individual or by combining them in pairs, with amplitude perturbation between 2000 and −2000 and step size of 100 (arbitrary units). This generated 1425 models. After removing highly distorted models, i.e. with more than 200 pairs of consecutive Cα atoms with distance artificially larger than 5 Å, we obtained a final number of 1415 models. Secondly, we used a Monte-Carlo protocol (38,39) to sample conformations based on combinations of the five lowest-frequency modes with random amplitudes. We adjusted the internal parameters to produce models with similar amplitude perturbations to those obtained by elNémo. To generate the same number of conformations, we initially built 3000 models, removed highly distorted models, and randomly selected 1415 models. Finally, we used iMC module from iMOD (40) to sample conformations by randomly selecting one of the five lowest-frequency modes in internal coordinates and its amplitude, in an iterative process. We applied the default parameters and produced the same number of models as the other two methods (1415) for the sake of consistency. The results from the three normal mode sampling methods produced a final set of 4245 Cα models, which were rebuilt by adding missing atoms and side chains and atomically refined with Modeller 8v1 (using the original Cα model as template) to fix incorrect bond distances (41,42).

### Structural modeling of translin–RNA complexes

We have devised here a new computational method for flexible docking of the single-stranded RNA to translin, based on the identification of possible dinucleotide binding sites on the translin surface to build different conformations of RNA bound to translin (Supplementary Figure S6). More specifically, we used FTDock v2.0 (43) to dock a rigid GU dinucleotide [modeled by MMB v2.3 (44)] to all the conformations found in the SAXS analysis of the free translin, as described in next sub-section and explained with more details in the Results section (as well as to the crystallographic structure, PDB code 1J1J). For the sake of efficiency, we selected only dinucleotide poses that were within 5 Å inter-atomic distance from any of the residues in the known nucleic acid binding equatorial region (R21, R36, R86, R92, Q96 and R192), as previously defined (28). The resulting GU docking poses were then clustered using a cutoff of 10 Å inter-atomic distance, and for each cluster, the GU docking pose with the best FTDock SCscore was selected as representative. All possible combinations of up to 12 consecutive dinucleotide docking poses were calculated using a graph-like algorithm, allowing “gaps” that would represent possible regions of the RNA molecule that would not be bound to translin. We clustered those combinations that were >70% identical (i.e. at least 9 out of the 12 dinucleotide docking poses were the same). Finally, for each representative combination, a helicoidal 5′-GUGUGUGUGUGUGUGUGUGUGUGU-3′ ssRNA molecule was modeled by MMB v2.3 (44) and refined by a quasi-Newton optimization protocol restrained to the C4' atom positions of the dinucleotide docked poses. This procedure resulted in a large initial list of 65 982 translin–RNA complex structures that included many impossible models with excessive number of atomic clashes with translin, as no specific conformation was imposed to the ssRNA when it was threaded to the docked dinucleotides. Therefore, we removed models with >150 atomic clashes and obtained a final list of 2865 translin–ssRNA conformations.

### Fitting structural models to SAXS data

The program CRYSOL (35) was used to calculate the theoretical SAXS curves for each of the full-length translin and translin–RNA models. Default parameters for CRYSOL calculations were used to derive theoretical profiles. All theoretical curves were computed with a number of 201 points and a maximum scattering vector of 0.4. For each octameric assembly, 10 different structures were computed by random addition of terminal tails to realistically cover the conformational sampling (see above). The individual theoretical curves were averaged and compared with the appropriate experimental SAXS curve, using χi as a figure of merit in a momentum transfer range of 0.020 < *s* < 0.40 Å^−^^1^.

In the case of free-translin, Minimal Ensemble Search (MES) (45) was used to find a minimum ensemble of conformations from the 4245 models built with NMA (in which terminal tails were modeled as explained above). In the case of translin–RNA complex, we calculated the SAXS profile for all generated translin–RNA models (see above), including the disordered N- and the C-terminal regions of translin, and evaluated which ensembles of conformational states provided the best fitting to the SAXS data, using MES for combinations of up to five conformational states (45). To further study the conformational heterogeneity of ssRNA within translin, we also used the program Ensemble Optimization Method (EOM) (36) for combinations of up to 50 conformational states (five different runs were performed with EOM allowing structure repetition).

## RESULTS

### Translin is a dimer of tetramers in solution

To clarify the 3D arrangement of translin in solution and the structural basis for its nucleic acids recognition, we have studied the structure of human translin by SAXS, which provides information about the overall size and shape of biomolecules in solution (46–48). Analysis of the low-resolution part of the SAXS curve using Guinier’s approximation (33) indicates that translin is a particle with a radius of gyration (R_g_) of 48.4 ± 0.4 Å in solution (Supplementary Figure S1). The pairwise distance distribution function, *p(r)*, as derived from the SAXS curve, presents a prominent peak at ∼58 Å indicating that translin adopts a globular structure in solution (Supplementary Figure S1). In addition, the *p(r)* function displays a smooth decrease to the maximum intra-particle distance, *D*_max_, of 160 ± 5 Å. The overall picture derived from the primary analysis of the raw data would favor the C4 octameric model found in the crystallographic analyses of translin and translin–TRAX complexes as well as in the EM study of translin–TRAX (17,18,25,26), but is not compatible with the C8 octameric ring model proposed in an earlier EM study of translin (22). We further confirmed this observation by fitting of the C4 and C8 structural models to the SAXS data. These calculations also indicated that only the C4 octameric translin found by X-ray crystallography (1J1J PDB) displayed a reasonable agreement to the SAXS experimental curve (Supplementary Figure S2 and Supplementary methods).

### Translin shows conformation variability

The above-described SAXS data clearly favor the compact translin C4 arrangement derived by X-ray crystallography (25,26). Nevertheless, despite this overall agreement, the fitting was not optimal (*χ_i_* = 7.19). Two major reasons for this suboptimal fitting can be hypothesized: (i) the highly flexible N-terminal histidine-tag and the C-terminal tail, of 12 and 11 residues, respectively, which are present in the sequence construct used for experiment, but missing in the X-ray structure; and (ii) a putative conformational change that, while keeping the octameric C4 arrangement, could substantially modify the overall shape of the translin complex as suggested by the EM structure of *Drosophila* translin–TRAX (18).

**Figure 1.**
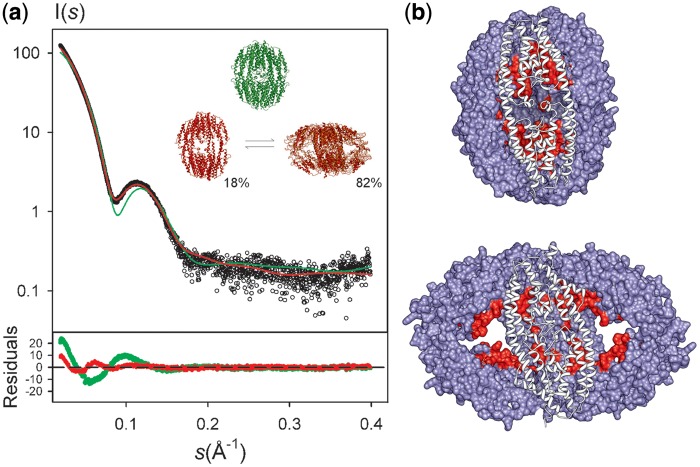
Structural models of translin according to SAXS data. (**a**) Experimental fit of the structural models of full-length translin (green) and best MES dynamic ensemble (red) to the experimental SAXS curve (black circles). The structure of the crystallographic translin yielded a fitting to SAXS data of *χ_i_* = 5.01. The best ensemble of translin conformers was found to be formed by a compact and an ensemble of three open conformations (with individual relative populations of 45.8, 23.3 and 13.3%), with a fitting to SAXS data of *χ_i_* = 1.67. Point-by-point deviations for each model are displayed at the bottom of the plot with the same color code. Although the full-length protein has been used in both fittings, flexible N-terminal histidine-tag and C- terminal regions are not shown here for the sake of clarity. (**b**) The RNA/DNA binding residues according to OPRA (49) prediction (previously validated in *S.Pombe* (28)) are shown in red for the two major configurations of translin.

The presence of disordered tails is compatible with the smooth decrease of *p(r)* towards the D_max_ value of 160 ± 5 Å. This hypothesis was tested by building models for full-length translin including the terminal tails (see ‘Materials and Methods’ section and Supplementary Figure S3). The averaged curve from an ensemble of 10 full-length translin conformers having the same global structure, but with terminal tails in random conformations, was compared with the experimental one (Figure 1a). Although the presence of the disordered tails improved the fit to the SAXS data, the goodness of fit value was not yet satisfactory (*χ_i_* = 5.01), suggesting that translin may adopt a global conformation in solution that differs from that in the crystal.

The global conformational space of translin was explored by NMA, a powerful technique that allows efficient conformational sampling in cases of large structural arrangements (50). We generated 4245 models of the translin octamer, and for each of them, a theoretical SAXS curve was computed by averaging the data derived from 10 different conformations of the full model including the N-terminal histidine-tag and C-terminal tail (‘Materials and Methods’ section and Supplementary Figure S4). The best fitting obtained by a single model was notably better than that obtained by the crystallographic structure (*χ_i_* = 2.24), but still far from optimal, suggesting that there could be conformational heterogeneity in solution. Therefore, an optimal combination of a small number of these models/curves that collectively describes the SAXS curve was obtained using the program MES (45) (see Materials and Methods). The conformational equilibrium found for translin notably improved the fitting to the SAXS curve (*χ_i_* = 1.67, see Figure 1a) by combining one compact conformation (18% population) and an ensemble of three open conformations (82% population). The compact conformation is very similar to the structure obtained by crystallography, but slightly more open in the longitudinal interdimeric region (Supplementary Figure S5). Open conformations present a maximum interparticle distance of 152.0 Å, in excellent agreement with the experimentally determined *D_max_*, 160 ± 5 Å. Interestingly, the open conformations found in this analysis leave the nucleic acid-binding residues accessible for interaction (Figure 1b).

### Site-directed mutagenesis supports the dynamic octameric model of translin

**Figure 2.**
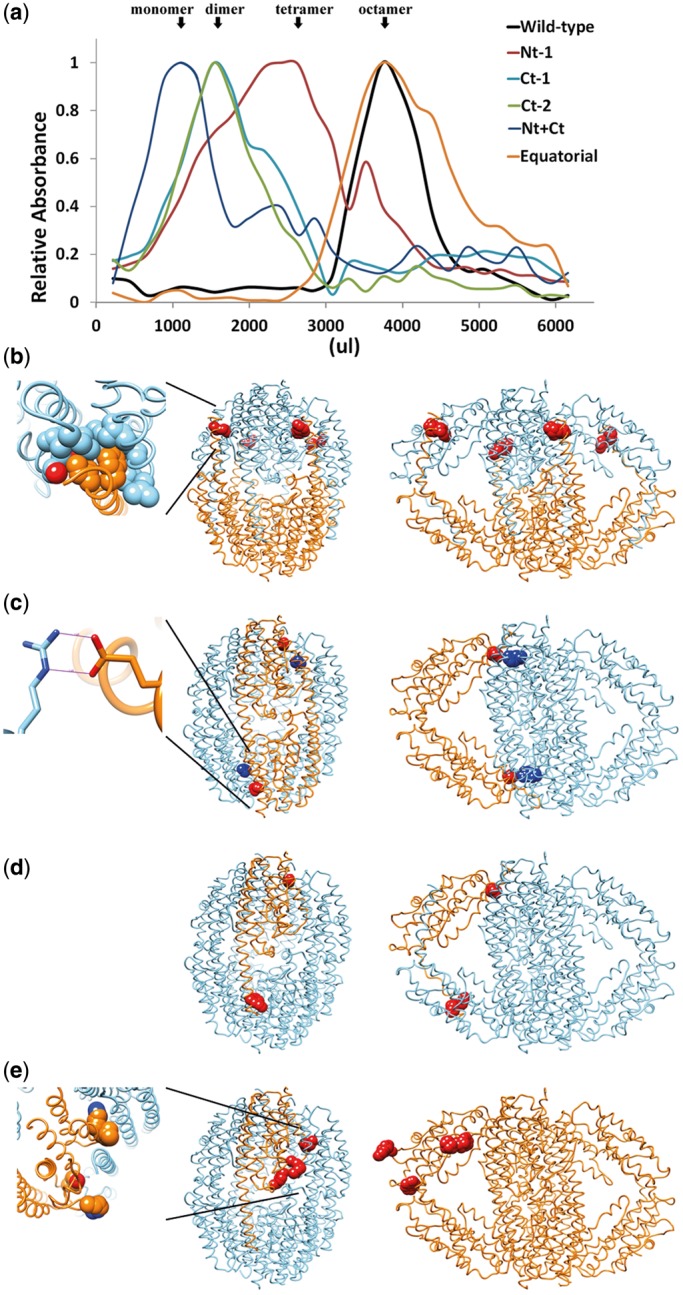
Glycerol gradient centrifugation of wild-type and mutant translin variants. (**a**) Sedimentation profiles of recombinant wild-type translin and the mutant translin variants listed in Table 1. Vertical arrows indicate the peaks of the major configuration of each of the translin mutant variants. (**b**) The two adjacent substitutions I6A and F7A of the mutant Nt-1 break the octamer in tetramers (a tetramer is shown in orange in the compact and open translin models, with the corresponding mutated residues in red CPK). The insert shows a hydrophobic pocket in which I6 and F7 of one monomer (in cyan) interact with the opposing residues of the second monomer (in orange). (**c**) The E206A substitution of Ct-1 mutant or the R153A substitution of Ct-2 break the octamer into dimers (one dimer is shown in orange in the compact and open translin models, with the corresponding mutated residues E206 in red CPK and R153 in blue CPK). The insert shows an ion bridge (indicated with two straight lines) between E206 in one monomer (in cyan) and R153 of the second monomer (in orange). (**d**) The Nt+Ct mutations (I6A, F7A and E206A substitutions) break the translin octamer into monomers (a monomer is shown in orange, with the corresponding mutated residues in CPK). (**e**) The *Equatorial* mutant (R182G/L183V/R129G/Y85A) has no effect in the octameric structure. Interestingly, according to the crystallographic structure, these mutants in a given monomer (in orange) are interacting with other monomers, while in the open conformation, these mutants are not located at the oligomer interface, which explains the incapability of this mutant to break up the octamer (see Discussion).

To further examine the proposed oligomerization model for translin, we generated a series of amino acid substitutions in the protein by site-directed mutagenesis, chosen according to the expected protein–protein interfaces in the different octameric models proposed for translin. These mutated translin variants were designated *Nt-1* (N-terminal 1), *Ct-1* (C-terminal 1), *Ct-2* (C-terminal 2), *Nt+Ct* and *Equatorial,* named according to the positions of the mutations in the monomer (Table 1; Figure 2). The translin protein variants encoded by these mutants were purified and their major oligomeric states were determined by ultracentrifugation in glycerol gradients (Figure 2a). It can be seen that while the wild-type translin sedimented as octamers, the major form of the protein variant Nt-1 sedimented as tetramers. It is also seen that the variants Ct-1 and Ct-2 sedimented as dimers, while the variant Nt+Ct sedimented as monomers.

**Table 1.** Summary of properties of wild-type translin and of translin variants

Translin	Mutations	S. value	MW (kDa)^a^	No. of subunits^b^
Wild type		9.11 ± 0.41	220.02	8 (8.2)
Nt-1	I6A/F7A	5.23 ± 0.24	90.83	4 (3.9)
Ct-1	E206A	3.88 ± 0.24	56.43	2 (2.1)
Ct-2	R153A	3.81 ± 0.27	55.41	2 (2.0)
Nt+Ct	I6A/F7A/E206A	2.77 ± 0.12	32.33	1 (1.2)
Equatorial mutant	Y85A/R129A,/ R182G/L183V	8.98 ± 0.52	215.24	8 (8.0)

^a^The molecular weights were calculated from the average S values as described in ‘Materials and Methods’ section.

^b^Estimates of the number of subunits (in brackets) were obtained by dividing the experimentally determined molecular weights of the complexes by the molecular weight of the human translin subunit (26.8 KDa). The actual number of subunits is also indicated. An octamer of translin has theoretical MW of 215 KDa.

Figure 2b–d shows the positions of these mutations at the interfaces of the open and closed octameric structures. Figure 2b shows the two substituted amino acids, I6 and F7 of each subunit in the variant Nt-1. These two hydrophobic amino acids in the α-helix 1 of each subunit interact with a cluster of hydrophobic amino acids in α-helices 1, 2, 3 and 4 of the adjacent subunit (see inset in Figure 2b). Hence, the substitutions of I6 and F7 by alanine were expected to cause disruption of this interaction and the separation of the two tetramers along the longitudinal axis, as observed. A similar representation of the E206A and R153A substitutions in the variants Ct-1 and Ct-2 is illustrated in Figure 2c, depicting a salt bridge formed between the residues E206 and R153 in two adjacent subunits (see inset in Figure 2c). Either of the two substitutions E206A or R153A disrupted this strong interaction, thereby causing the separation of dimers along the transversal axis. As expected, combining Nt-1 and Ct-1 substitutions in the Nt+Ct variant led to disruptions along both axes and produced monomers (Figure 2d).

Finally, the *Equatorial* four-mutant construct (Y85A/R129A/R182G/L183V) harboring changes along the equatorial axes of the monomer did not disrupt the octameric arrangement (Figure 2a). Interestingly, these substituted amino acids are (albeit weakly) involved in the interactions between the subunits in the X-ray-based octameric structure (Figure 2e), but they are not at all involved in the interactions between subunits in the open conformations. Thus, the observation that these equatorial mutations did not disrupt the octameric arrangement is consistent with the existence of highly populated open conformations in solution.

### RNA is bound to the central cavity of the dynamic translin assembly

To identify the structural features of RNA recognition by translin, SAXS data were obtained for a translin–RNA complex formed by the mixing of translin and the oligoribonucleotide (GU)_12_, which has high affinity for translin (*K*_d_ = 2 nM) (8,29). The SAXS curve measured for this complex displayed similar features to that of the free translin, indicating that the overall arrangement is not changed upon ssRNA binding. The nucleoprotein complex presents a R_g_ value of 47.2 ± 0.3 Å that is slightly smaller than that found for free translin, 48.4 ± 0.4 Å (Supplementary Figure S1). The *p(r)* function derived from the SAXS curve of the complex presents an equivalent D_max_ value to that found for free translin, 160 ± 5 Å, indicating that similarly open conformations are present in the complex. The *p(r)* function of the complex presents a maximum value at a slightly smaller radius, 56 Å, than that of free translin, 58 Å. This last observation and the smaller R_g_ value found in the complex can be explained if the ssRNA is placed in the interior of the translin cavity, partially filling the hollow sphere that represents the protein (46).

**Figure 3.**
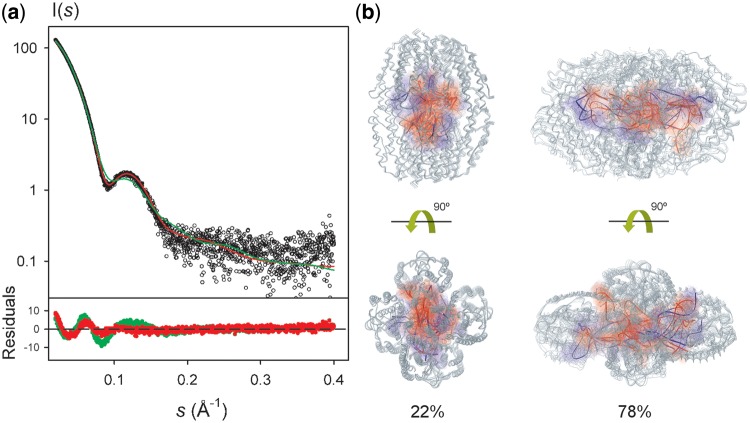
Structural and dynamic models of translin–ssRNA complexes according to SAXS data. (**a**) Experimental fit of the best single-conformation of the complex (in green) and the dynamic structural model of translin–RNA interaction (in red) to the experimental SAXS curve (black circles). Point-by-point deviations for each model are displayed at the bottom with the same color code. (**b**) The best equilibrium of conformers was found to be formed by an ensemble of compact and open translin forms bound to structurally different RNA molecules, with a fitting to SAXS data of *χ_i_* = 1.62, notably better than the one found for the best single conformation (*χ_i_* = 2.44). Translin is shown in gray ribbon, while the docked RNA conformations are shown in ribbon and surface, colored according to their population within the global ensemble (red is higher population; blue is lower). Two different views are displayed.

To have a more detailed picture of the translin–RNA complex, we generated putative structural models by applying an innovative procedure for flexible ssRNA docking. The X-ray structure of translin and the four conformations derived from the SAXS analysis of the free translin were used as receptors to dock rigid GU dinucleotides that were subsequently connected using a graph-like algorithm to create 2865 models of the nucleoprotein complex (see Materials and Methods and Supplementary Figure S6 for details). We tested the capacity of each one of these models to describe the experimental curve. The best fit obtained (χ_i_ = 2.44) is displayed in Figure 3 and presents systematic departures from the experimental curve in a broad momentum transfer range. The incapacity to describe the experimental curve with a single conformation prompted us to use ensemble methods. Using MES as optimization tool, several ensembles of translin–RNA complexes were found with a similarly good agreement to the SAXS data (χ_i_ = 1.73–1.75) (Supplementary Figure S7). In all these ensembles, the relative population of compact (10–16%) and open (84–90%) translin forms was similar to that in the free translin, consistent with the equivalent D_max_ values found in both cases (*D*_max_, 160 ± 5 Å). These values are also in excellent agreement with the maximum interparticle distance of 152.8 Å calculated for the open translin forms in these complexes. The ssRNA molecules in these complexes adopted many different conformational states (Supplementary Figure S7). However, it is not possible to disregard that such conformational heterogeneity might arise from the low-resolution nature of the SAXS technique, which does not allow distinguishing between some of the different RNA conformers generated during the docking process. To further explore the conformational heterogeneity of bound RNA, we evaluated the fitting to the SAXS curve by using the EOM, which allows the consideration of larger ensembles of coexisting structures (up to 50) (36). An excellent agreement to the experimental curve was obtained (χ_i_ = 1.62), and the relative population of open (22%) and compact (78%) translin forms was similar to that previously found with MES for the complex and the free states (Figure 3a). The ssRNA molecules found in the optimized ensemble showed a large conformational heterogeneity, which is compatible with a model in which the RNA molecules do not adopt a fix configuration when placed within the translin cavity (Figure 3b). In a scenario where translin displays a high level of plasticity, an intrinsically flexible molecule such as ssRNA, which is composed of a repetitive sequence and lacks a localized recognition region, most probably may adopt multiple configurations within a symmetric cavity hosting multiple binding sites.

## DISCUSSION

### Translin is a highly flexible assembly in solution

Our results clearly demonstrate that translin forms a highly plastic octameric assembly in solution. This flexibility was already suggested by the absence of conserved hydrogen bonds between the two tetramers in the crystallographic structure of human translin (26). Here, we present SAXS data that actually indicate the coexistence of two major arrangements of translin octamers in solution: open and compact. The open state constitutes ∼82% of the octamers population, whereas the compact state only represents ∼18% of the octamers. We should note here that the presence of residual interparticle interactions not completely suppressed by the data processing (see Methods) could entangle the exact determination of the relative population of both families of conformations, causing a slight overestimation of the fraction of molecules in the open conformation. The compact form is structurally similar to the crystallographic structure, indicating that during the crystallization process the more symmetrical and compact form is selected despite not being the major species in solution.

The conformational changes of translin involve the loss or gain of most of the dimer–dimer interactions, which suggests that these are formed by weak intermolecular contacts. This model explains why our *Equatorial* variant remains octameric, as the mutated residues are not involved in dimer–dimer contacts in the open state (Figure 2e). In contrast, strong interdimeric interactions occur in the N-term and C-term regions of translin in both the open and compact models. These contacts maintain the structure of the global assembly while allowing the conformational dynamics of the system. As a consequence, mutations in these regions cause disassembly of the translin octamer into different oligomeric states (Figure 2b–d).

These observations regarding the inherent plasticity of translin are consistent with the Debye–Waller factors (B-factors) obtained in the crystallographic refinement of translin (PDB 1J1J) (26), which displays an inhomogeneous behavior along the sequence. Interestingly, residues in the equatorial region of the structure, located at the hinge of the dimeric units, present larger B-factors, suggesting an increased degree of mobility in comparison with residues in the polar regions (Supplementary Figure S8). The conformational fluctuations found in the crystallographic environment are compatible with the necessity of flexibility in those regions, which allows the transition between the open and compact translin conformations in solution. It remains to be seen whether structurally homologous systems, like translin–TRAX octameric complex, also have the same alternative conformational states in solution as pure translin octamer. In view of the high homology of translin and TRAX, one would expect similar structural architecture. However, the presence of one or several catalytic TRAX monomers at different positions within the translin octamer might modify the equilibrium population of conformers and hence the binding affinity for nucleic acids and the catalytic activity, perhaps tuning the dynamics and the biological activity of the assembly while keeping the overall architecture.

### Conformational heterogeneity of nucleic acids binding to translin

Translin has been shown to bind ssRNA and ssDNA in solution (2,7–9,29), which has been linked to possible roles in mRNA translation and transport, RNAi metabolism (2,12,15,17,18,51,52) and tRNA processing (19), as well as in other possible functions of DNA metabolism (7–9). Remarkably, while the previously identified nucleic acid-binding region (28) is not accessible for nucleic acids binding in the compact (crystallographic) structure, it becomes accessible in the open configurations of translin in solution found here based on SAXS data (Figure 1b). This suggests that the binding of translin to nucleic acids is related to the conformational equilibrium of the protein in solution, in which the open conformations could facilitate the process of recognition. Interestingly, the dimensions of the groove in the open conformations (length ∼60 Å; width ∼25 Å) would be too tight for accommodating a double-stranded DNA molecule, in agreement with the experimentally observed inability of translin to bind double-stranded DNA (8). According to the similarity of the *p*(r) functions derived from the free and bound states (Supplementary Figure S1), the presence of ssRNA does not rigidify translin. The repetitive sequence of the intrinsically flexible ssRNA (GU)_12_ that hosts several binding regions to translin, and the presence of multiple and symmetric binding sites within translin cavity produces a multiplicity of potential low-affinity interactions that do not perturb the inherent breathing of translin. This observation is coherent with the large conformational variability of ssRNA that was found to be compatible with the SAXS data. Based on these considerations, we speculate that the translin octamer conformational dynamics might be essential for the accommodation of different RNA or DNA molecules, and such model could provide an explanation for the differential affinities of the protein for specific RNA and DNA sequences (8,29).

### Implications for the biological functions of translin and TRAX proteins

Drosophila and human translin–TRAX complexes were found to have an endonucleolytic activity, which degrades the ago2-nicked passenger RNA strand in RNA-induced silencing complexes (RISC), thereby activating those RISC complexes (15,17,18). In *Neurospora* such complexes appear to be involved in the removal of 5′ pre-tRNA fragments after the processing of pre-tRNAs by RNase P (19). The catalytic sites for these degradation activities reside in the TRAX subunits. Although the structural studies reported in this article were all carried out with pure recombinant translin complexes and the presence of TRAX could introduce subtle changes in the dynamics of the translin–TRAX octamers (as discussed above), it appears reasonable to think that the overall transitions will be the same, and it would be interesting to explore in the future whether such transitions could have a role in the endonuclease activity of the system. It should also be noted that pure translin octamers can be formed in cells in addition to translin–TRAX complexes. Translin octamers could be engaged in other functions that have been previously suggested, such as participation in specific mRNA trafficking along dendrites of nerve cells (52). In fact, it appears that activities such as RNA transport might be carried out by pure translin octamers, given that they can bind RNA, but lack the endonuclease activity of TRAX. In this way, the cargo RNA will be maintained intact during the translocation activity. Furthermore, the localization of RNA in the internal cavity of pure translin complexes (as shown in this article), could protect the transported mRNA from being translated in the inappropriate location, or from being degraded by other nucleases, which would help to maintain its functionality (51). Interestingly, only TRAX has a nuclear localization signal, while translin has just nuclear export signal (4,5,53). Thus, pure translin octamers would be expected to remain in the cytosplasm and carry out cytoplasmic functions such as mRNA trafficking in dendrites. In summary, the inherent plasticity of translin assembly shown in this work provides a structural basis for its diverse biological roles.

## SUPPLEMENTARY DATA

Supplementary Data are available at NAR Online, including [54–58].

## FUNDING

FPU fellowship from the Spanish Ministry of Science (to L.P.-C.); fellowship from the Clore Foundation PhD Scholars program (to K.L.); Spanish Plan Nacional I+D+i [BIO2010-22324]; SPIN-HD—ANR Chaires d’Excellence from the Agence National de Recherche; Israel Science Foundation [967/08]. Funding for open access charge: Spanish Plan Nacional I+D+i (BIO2010-22324) and the SPIN-HD—ANR Chaires d’Excellence from the Agence National de Recherche.

*Conflict of interest statement*. None declared.

## Supplementary Material

Supplementary Data
