# Supplementary material for: Conformational transitions in human translin enable nucleic acid binding
Source: Nucleic Acids Res. 2013 Aug 26;41(21):9956–66. doi: 10.1093/nar/gkt765 (PMC3834833; doi:10.1093/nar/gkt765)
Supplement: Supplementary Data [file supp_41_21_9956__index.html]

Conformational transitions in human translin enable nucleic acid binding — Supplementary Data 

# Conformational transitions in human translin enable nucleic acid binding

## Supplementary Data

files

**Files in this Data Supplement:**

- Supplementary Data - pdf file
