# Supplementary material for: Conformational transitions in human translin enable nucleic acid binding
Source: Nucleic Acids Res. 2013 Aug 26;41(21):9956–66. doi: 10.1093/nar/gkt765 (PMC3834833; doi:10.1093/nar/gkt765)
Supplement: Supplementary Data [file supp_gkt765_nar-00531-h-2013-File005.pdf]

Supplementary data

Methods

Building of translin EM-based octameric ring models

Firstly, an ensemble of models was generated by sampling geometrically valid configurations of translin octameric rings using Multifit (54), with a single subunit of human translin (PDB code 1J1J, chain A) and an EM density map at 18 Å resolution (22) as input. The MultiFit run resulted in 3651 models. Secondly, due to the low resolution of the density map, we hierarchically clustered the models, using the unweighted average distance criteria, into 944 clusters, such that the maximum global Cα RMSD within a cluster was 8 Å. The number of models in each cluster ranged between 1 and 32. Representative solutions from all clusters were ranked according to cross-correlation fitting score and those with a fitting score lower than 0.5 were excluded. The resulting 93 cluster-representatives were further filtered, by clustering models with less than 10 Å of Cα RMSD between monomers (i.e. superimposing monomer to monomer, and calculating RMSD for each of the other monomers), keeping as representative of each cluster the model with best fitting score. Then, we evaluated the 30 representative models considering different physico-chemical and biological criteria such as: *i)* cluster size; *ii)* fitting score; *iii)* pyDock binding energy of the octamer (55); *iv)* percentage of the ODA residues (56) located at the interface; *v)* accessibility of nucleic acid binding region (28); *vi)* EM-based (22) diameter of the ring and central regions ($Ø_{min}$ = 30 ±10 Å, $Ø_{central}$ = 50 ±10 Å, $Ø_{max}$ = 115 ±10 Å); and *vii)* involvement of L184 and L191 residues in monomer-monomer interfaces (57). Finally, we selected the two models that best satisfied all these criteria. We also refined the models with SymmRef (58) in order to account for possible conformational changes, but the results did not change.

54. Lasker,K., Topf,M., Sali,A. and Wolfson,H.J. (2009) Inferential optimization for simultaneous fitting of multiple components into a CryoEM map of their assembly. *J. Mol. Biol.,* **388(1)**, 180-194.

55. Cheng,T.M., Blundell,T.L. and Fernandez-Recio,J. (2007) pyDock: electrostatics and desolvation for effective scoring of rigid-body protein-protein docking. *Proteins,* **68**, 503-515.
56. Fernández-Recio,J., Totrov,M., Skorodumov,C. and Abagyan,R. (2005) Optimal Docking Area: a new method for predicting protein-protein interaction sites. *Proteins,* **58**, 134-143.
57. Aoki,K., Suzuki,K., Ishida,R. and Kasai,M. (1999) The DNA binding activity of Translin is mediated by a basic region in the ring-shaped structure conserved in evolution. *FEBS Lett.,* **443(3)**, 363-366.
58. Mashiach-Farkash,E., Nussinov,R. and Wolfson,H.J. (2011) SymmRef: a flexible refinement method for symmetric multimers. *Proteins,* **79(9)**, 2607-2623.

Figures

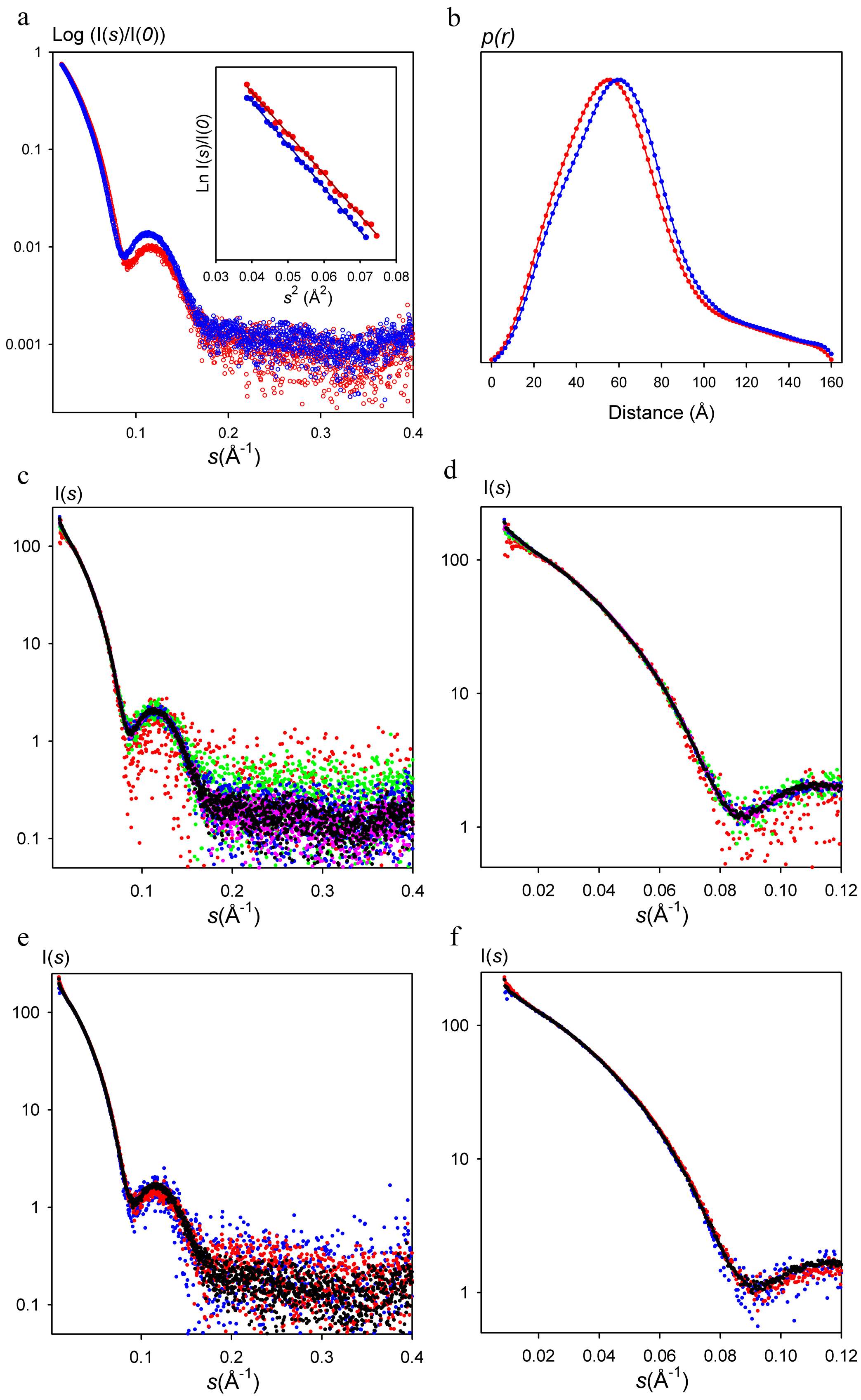

a
Log (I(s)/I(0))
1
0.1
0.01
0.001
0.1
0.2
0.3
0.4
s(Å^{-1})
Ln I(s)/I(0)
0.03
0.04
0.05
0.06
0.07
0.08
s^2 (Å^2)
b
p(r)
0
20
40
60
80
100
120
140
160
Distance (Å)
c
I(s)
100
10
1
0.1
0.1
0.2
0.3
0.4
s(Å^{-1})
d
I(s)
100
10
1
0.02
0.04
0.06
0.08
0.10
0.12
s(Å^{-1})
e
I(s)
100
10
1
0.1
0.1
0.2
0.3
0.4
s(Å^{-1})
f
I(s)
100
10
1
0.02
0.04
0.06
0.08
0.10
0.12
s(Å^{-1})

Figure S1. SAXS data of translin in solution. (a) SAXS curves and (b) pairwise distance distribution functions, *p(r)*, calculated from the SAXS curves of the free translin (blue) and its complex with $(GU)_{12}$ (red) using the program GNOM (34). The inset in panel (a) displays the region of the curve used for the derivation of the R_g and the *I(0)* values using Guinier's approach. The same colour code was used. The gaussian shape of *p(r)* indicates that both translin and the complex with ssRNA appear like a spherical-like particle in solution. A slight displacement of the maximum of the *p(r)* function towards smaller radii can be observed for the translin-ssRNA. The GNOM derived R_gs are 48.2 Å and 47.3 Å for translin and translin-ssRNA, respectively. These values are in very good agreement with the Guinier's derived ones, 48.4 Å and 47.2 Å, respectively. (c) SAXS curves from the dilution series measured for translin at 5.9 (black), 4.1 (purple), 3.0 (blue), 1.5 (green) and 0.8 (red) mg/ml, and (e) translin:ssRNA complex at 5.9 (black), 3.0 (red) and 1.5 (blue) mg/ml. The lower-angle region is also shown in greater detail (d, f).

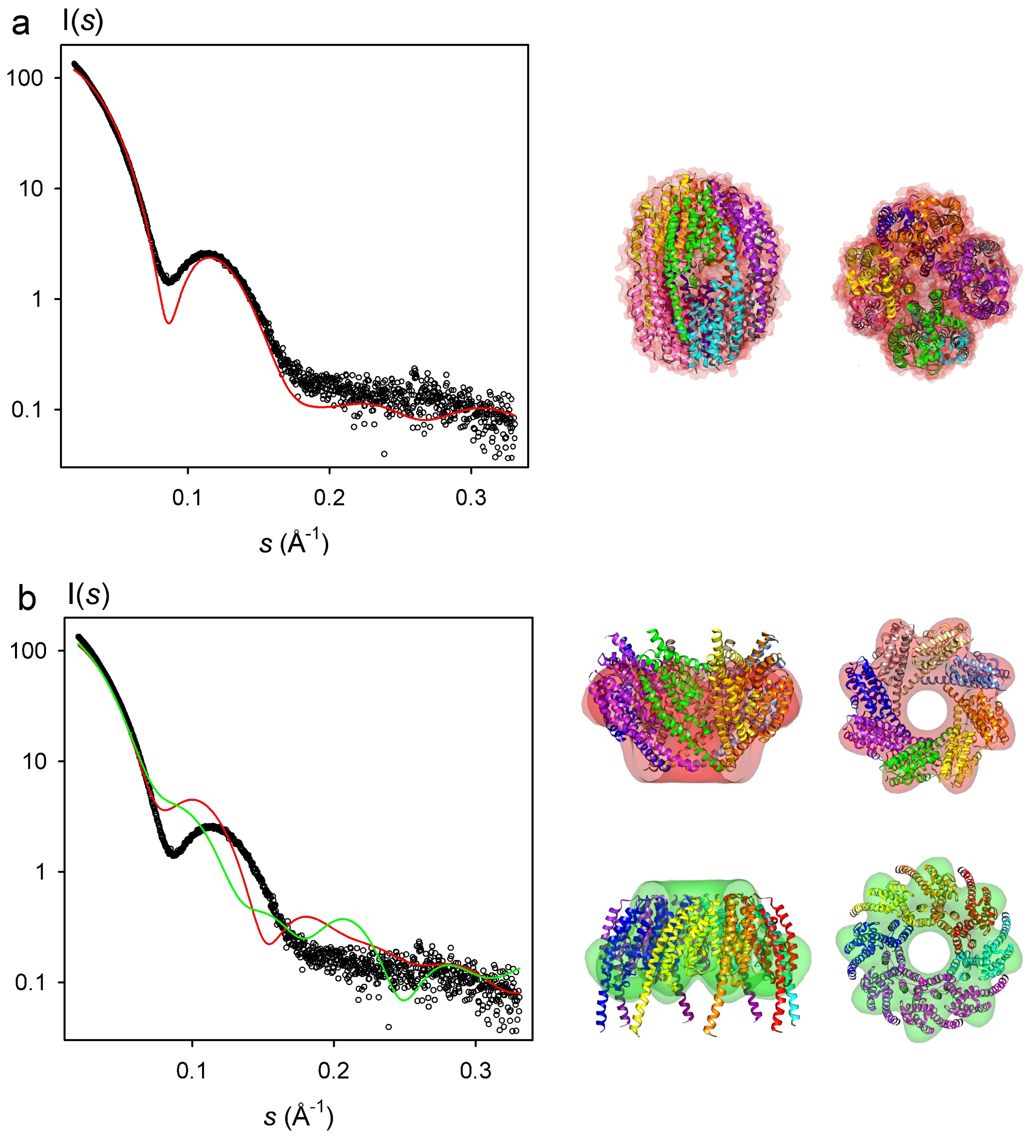

Figure S2. Fitting of the x-ray crystallographic structure and the best octameric ring models to the SAXS curve. (a) Fit of the crystallographic C4 translin octamer (red line) to the SAXS curve (black circles). The octamer structure is shown at the right panels in different orientations. (b) Fit of the models derived from translin EM to the SAXS curve. The first (green line) and second (red line) scored models according to several energetic and experimental criteria (detailed in SI Text) are shown. The structure corresponding to each model is shown at the right panel in two different orientations, with the EM map coloured in green and red, according to its corresponding fitting curve in the SAXS panels on the left.

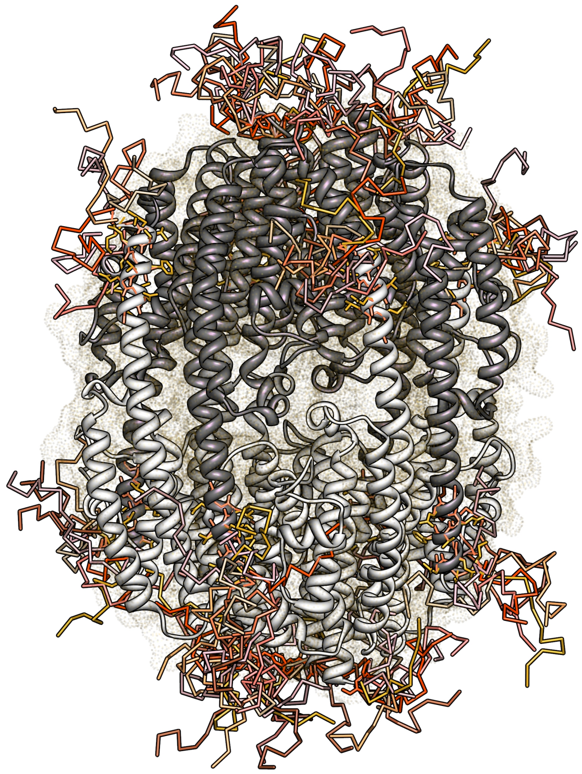

Figure S3. Conformational ensemble for C-term and N-term unstructured regions. The x-ray structure of translin (1J1J PDB) is shown in grey ribbon and transparent surface. Ten modeled N- and C-terminal regions are shown in stick, coloured in different brown levels.

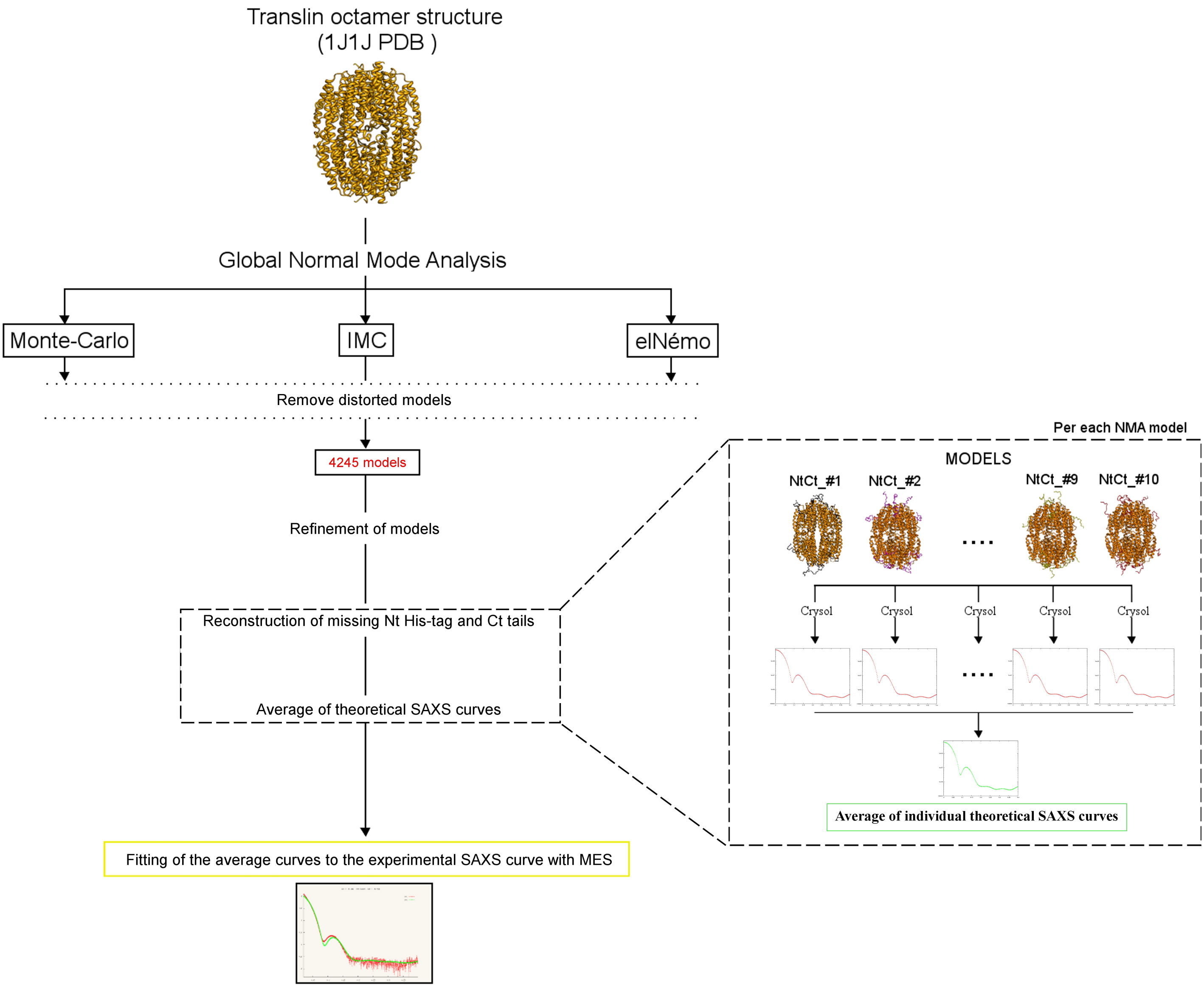

Figure S4. Scheme of the translin conformational search and fitting to SAXS procedure.

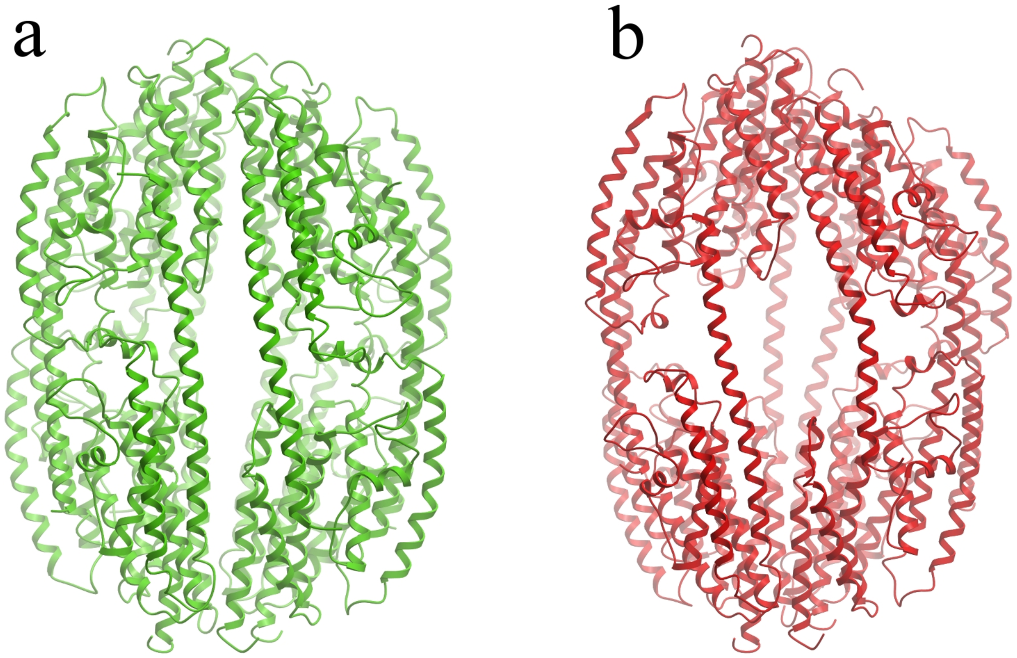

Figure S5. Comparison of translin compact conformations. (a) Crystallographic translin structure (1J1J PDB). (b) Compact state found by NMA and selected by fitting to SAXS data (as part of a 4-state equilibrium).

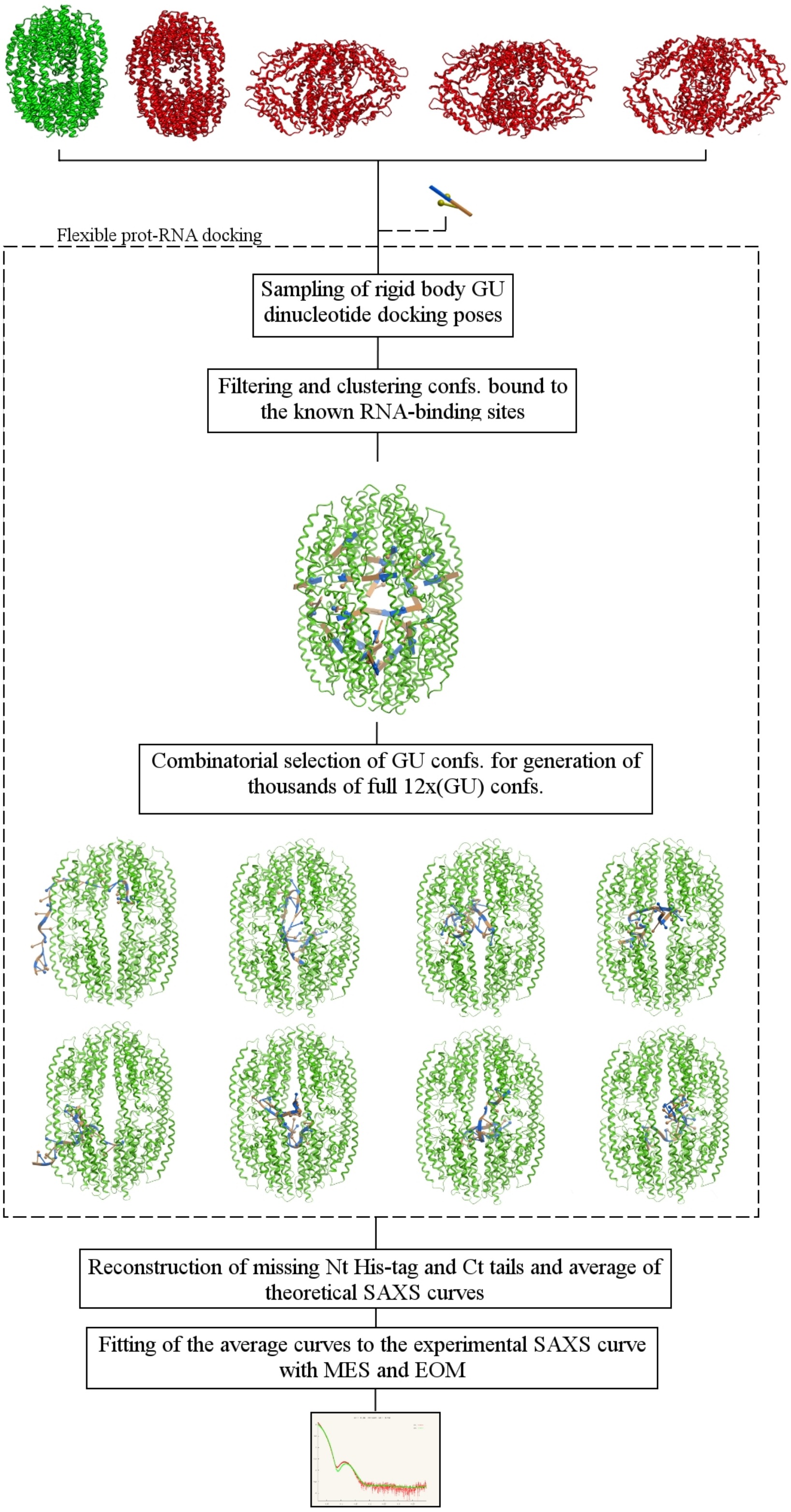

Figure S6. Scheme of the translin-RNA conformational search and fitting to SAXS procedure.

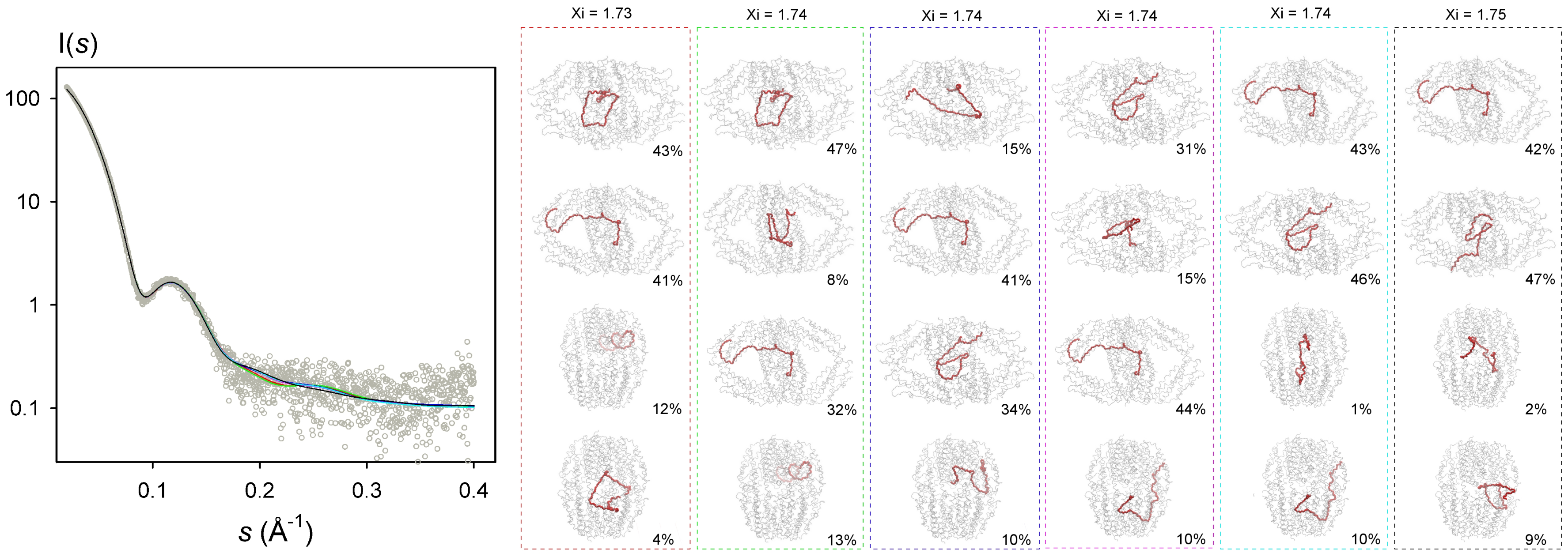

I(s)
100
10
1
0.1
0.1
0.2
0.3
0.4
s (Å-1)
Xi = 1.73
43%
41%
12%
4%
Xi = 1.74
47%
8%
32%
13%
Xi = 1.74
15%
41%
34%
10%
Xi = 1.74
31%
15%
44%
10%
Xi = 1.74
43%
46%
1%
10%
Xi = 1.75
42%
47%
2%
9%

Figure S7. Different combinations of translin-RNA conformers yield similar fitting to SAXS. We show six combinations of four translin-RNA conformers that yielded good fitting to SAXS data using MES. Each conformational equilibrium set is framed by a square, colored according to the corresponding fittings. Similar relative populations of translin conformers (ranging from 84% to 90% for open conformations and from 10% to 16% for the compact ones) are bound to different RNA conformations, while giving similar fitting values, suggesting conformational heterogeneity for RNA binding.

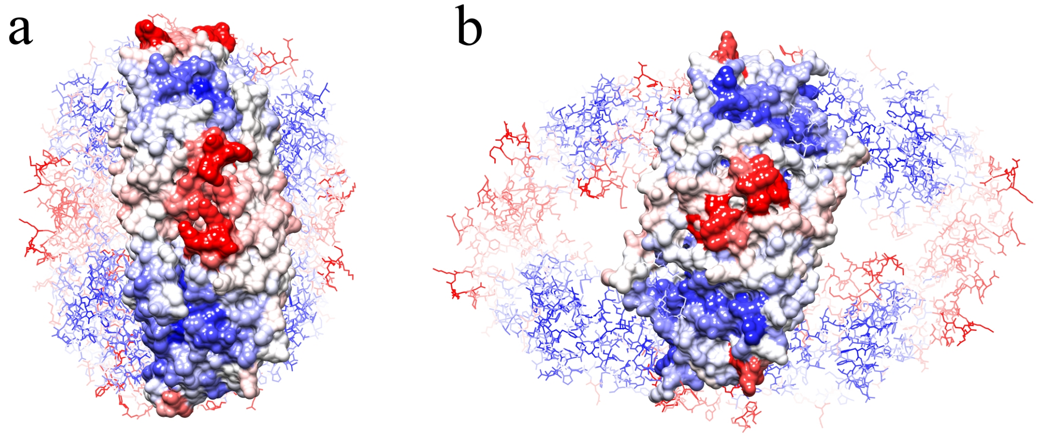

Figure S8. Crystallographic B-factor values per residue. (a) Compact and (b) open translin structures, with residues coloured according to the B-factors derived from the crystallographic structure (blue for lower B-factors; red for higher B-factors). Large B-factor values are observed in the hinge regions that facilitate the opening of translin.
